# Childhood cancers: what is a possible role of infectious agents?

**DOI:** 10.1186/1750-9378-8-48

**Published:** 2013-12-10

**Authors:** Kenneth Alibek, Assel Mussabekova, Ainur Kakpenova, Assem Duisembekova, Yeldar Baiken, Bauyrzhan Aituov, Nargis Karatayeva, Samal Zhussupbekova

**Affiliations:** 1 School of Science and Technology, Nazarbayev University, 53 Kabanbay Batyr Avenue, Astana 010000, Kazakhstan; 2 Nazarbayev University Research and Innovation System, 53 Kabanbay Batyr Avenue, Astana 010000, Kazakhstan; 3 National Medical Holding, 2 Syganak Street, Astana 010000, Kazakhstan

**Keywords:** Childhood cancer, Herpesviruses, Leukemia, Lymphoma, CNS, Infection, Papillomaviruses, Polyomaviruses

## Abstract

The etiology of childhood cancers has been studied for more than 40 years. However, most if not all cancers occurring in children are attributed to unknown causes. This review is focused on the role of infections in cancer development and progression in children. The main infectious agents include human herpesviruses, polyoma viruses, and human papilloma viruses. It is known that infections can lead to carcinogenesis through various mechanisms, and most likely act in addition to genetic and environmental factors. Given the importance of the infectious etiology of childhood cancers, clinical implications and possible prevention strategies are discussed.

## Introduction

Cancer is the second leading cause of death in children under 15 years of age. Childhood cancers (CC) include a variety of malignant tumors. Annual incidence worldwide is approximately 160,000 cases per year, whereas mortality rates average 90,000 [1]. Leukemia is diagnosed in about 30 − 34% of all CC [1]. Acute lymphoblastic leukemia (ALL) and acute myeloblastic leukemia (AML) are the two most frequent types of leukemia found in children [1]. The former occurs approximately five times more frequently than the latter, and accounts for approximately 75% of all childhood leukemia cases [1]. Central nervous system (CNS) cancers are the second most frequent in pediatric oncology, accounting for about 27% of CC [2]. The occurrence of CNS tumor types in children differs by type, with 30% low-grade glioma, 25% medulloblastoma, 20% high-grade glioma, 10% atypical teratoid/rhabdoid tumor, and 15% other CNS tumors [3]. Lymphomas represent the next most common type of CC, averaging 15% [4]. According to the National Cancer Institute, Hodgkin’s lymphoma (HL) accounts for 4 − 6% of all childhood cancers, with the highest incidence rates in 15–19 year olds [2]. Non-Hodgkin’s lymphomas (NHL) account for 6 − 7% of all childhood malignancies, and are comprised of the four most frequent subtypes, Burkitt’s lymphoma (BL), lymphoblastic lymphoma (LL), diffuse large B-cell lymphoma (DLBCL), and anaplastic large cell lymphoma (ALCL) [4]. There are other subtypes of childhood NHL, but they account for less than 5% of all cases. Incidence statistics for soft tissue cancers is around 9%, whereas for bone cancers (including osteosarcoma and Ewing’s sarcoma) the incidence is 6% [2]. Neuroblastoma is diagnosed in 7% of the CC cases, nephroblastoma (Wilms) tumors occurs in 5% and retinoblastoma in 3% CC cases [2].

Cancers found in children are usually quite distinct from those seen in adults. Because the causes of childhood cancers are unknown, it is difficult to determine a specific mechanism. This may be due to different exposures to environmental hazards/infectious agents coupled with immature physiological systems, as well as vulnerability at critical developmental stages [5]. In this review, we address the possible role of infectious agents in the onset and progression of childhood tumors.

Our search strategies included a background literature review, then systematic analysis and all-round discussion of found information on childhood cancers, risk factors and mechanisms of infectious agents. General and advanced search via the most comprehensive scientific databases on life sciences and biomedical topics, such as MEDLINE and SpringerLink, were used to conduct effective literature review. The search specifications narrowed species to “Human”, language to “English” and article types to “Review, clinical, trials, meta-analyses, and case reports”. Reference lists were thereafter hand-searched for additional articles. Then the full text articles were obtained and analyzed for actuality and authenticity of information.

### Possible causes and risk factors

To date, in addition to inherited factors, the World Health Organization (WHO) classifies four different groups of external agents as carcinogens which cause cancer in children. These are physical, biological, chemical carcinogens, and dietary components (e.g., cured meats) [5].

Extensive data is now available on known and suspected risk factors for childhood cancers, including but not limited to: early-life exposures to infectious agents (viruses, bacteria, protozoa, and fungi); parental, fetal, or childhood exposures to environmental toxins (pesticides, solvents, and household chemicals); parental occupational exposures to radiation or chemicals; parental medical conditions during pregnancy or before conception; maternal diet during pregnancy; early postnatal feeding patterns and diet; maternal reproductive history, and familial and genetic susceptibility; and risk associated with exposure to HIV[5]. Genetic risk factors usually include familial aggregations of genetic syndromes such as retinoblastoma, Li-Fraumeni syndrome, hereditary nonpolyposis colon cancer, ataxia telangiectasia, and others [6].

However, there are still no definitive causes identified for CC. In general, in 5 − 15% of CC cases genetic factors are thought to predispose the child to the development of cancer [7]. Environmental and exogenous factors have much lower figures (5 − 10%), leaving the vast majority of CC (75 − 90%) poorly understood and of unknown causes [8,9]. Because cancer is a multifactorial disease caused by genetic and environmental factors, it is often difficult to determine the critical period of exposure as during pregnancy or earlier [9]. In addition, childhood cancers develop and manifest differently from one another due to multiple numbers of causes and distinctive clinical courses with respect to age, ethnicity, and gender [10].

### Role of infections in etiology of childhood cancers

#### Leukemia

Two models have been proposed to explain how infectious agents could play a role in the development of childhood leukemia. The first model relies on the direct transforming ability of transforming viruses. Secondly, the effect might be due to the problems caused by abnormal immunological responses to congenital, neonatal, or post-neonatal infections, which in turn promote secondary genetic or immunological alterations. In this case, the action of microorganisms may be indirect and non-transforming (reviewed in [11]). One of the main agents in the proposed infectious association with childhood leukemia is a group of human herpesviruses, especially Epstein-Barr virus (EBV) and human herpesvirus 6 (HHV-6).

Associations of EBV with childhood leukemia, mainly lymphoblastic leukemia (ALL), have been found in some seropositivity studies, genetic analyses, and epidemiological studies. Initially it was shown [12] that EBV viral capsid antigen (VCA) IgM, in EBV-seropositive mothers, was associated with increased risk of acute ALL in offspring. In addition, Z Epstein-Barr replication activator (ZEBRA) IgG and VCA IgM antibody associations were attributed to increased risk of non-ALL in offspring. However the statistical significance of the results was not confirmed by a larger study of the same group [13]. The presence of VCA antibodies is recognized as a sensitive measurement for active infection. While for EBV reactivation, testing for ZEBRA and for EBV early antigen antibody was found to be useful. In addition, ZEBRA IgG and VCA IgM antibody associations were attributed to increased risk of non-ALL in offspring. EBV ZEBRA protein interacts with mitotic chromosomes, and with p53 and promyelocytic leukemia proteins. This might suggest a proposed mechanism for the transforming activity of the virus. Recent DNA studies show that latent membrane protein 1 of the EBV gene transcripts was found in 29/80 of the cancer cases versus 0 in the control group, in cases of ALL, AML, and chronic myelogenous leukemia (CML) [14]. ZEBRA protein transcripts and active EBV replication was detected by PCR and western blot in ALL patients. In the study positive controls (patients with Burkitt’s lymphoma and infectious mononucleosis) were consistently positive, but healthy donors, other disease controls, cases of AML and multiple myeloma showed negative results [15].

There are several mechanisms by which EBV could increase the risk of malignant transformation of infected cells. It was found that viral proteins inhibit apoptosis [16-18], affect the JAK/STAT pathway [19-22], promote epigenetic changes [23-25], and undermine the immune defense mechanisms [26] (see Table 1 for details).

**Table 1:** Possible carcinogenic mechanisms of viruses implicated in childhood cancers

**Virus and its product**	**Mechanism**	**Reference**
	**Inhibition of apoptosis**	
- EBV		
➢ EBNA-1	- Related to BCL2	[16]
➢ BHRF1, BALF1	- Related to BCL2	[17]
➢ EBNA3A, 3C	- Inhibit Bim	[18]
- CMV		
➢ IE1, IE2	- Inhibit apoptosis by activating PI3K pro-survival pathway	[27,28]
➢ UL36	- Confers resistance to chemotherapy in neuroblastoma	[29]
	**Disruption of signaling pathways/autonomous growth**	
- HHV-6 (U95)	- U95 binds NF-kB, probably deregulating the pathway	[30]
- EBV	- JAK/STAT pathway implicated L6 released by macrophages surrounding nasopharyngeal epithelial cells, binds to its receptor and activates STAT3, which triggers transcription of its target genes (cyclin D1, Bcl-xL, c-myc, survivin, VEGF). A correlation of VEGF expression as a key angiogenic factor in NPC metastasis is supported.	[19-21]
		[22]
- SV40	- Activation of growth factors and autocrine growth of mesothelioma cells, while RASSF1 is inactivated	[31]
	**Ablation of tumor-suppressors p53: viral proteins bind and thereby inactivate the tumor suppressors**	
- HPV 16/18 (E6, E7)	- Bind and inactivate p53	[32]
- BKV	- BKV is present in neuroblastomas, and colocalizes to p53.	[33]
- HHV-6 (U14 and ORF-1)	- Bind and inactivate p53	[30]
	**Promotion of epigenetic changes/RNA interference**	
- EBV	- EBV-infected cells acquire extensive methylation to silence multiple tumor suppressor genes.	[23]
➢ LMP1	- LMP1 downregulates CDH1 0.2-fold through upregulation of DNMT1, DNMT3A, DNMT3B, and 3-8-fold in NPC076 NPC cell line.	[24]
➢ miR-BART5	- Downregulation of PUMA expression in EBV-positive NPC and gastric carcinoma cells; decreasing levels of miR-BART5 or expression of PUMA can revert the suppression of apoptosis.	[25]
	**Suppression and evasion of the immune system**	
- CMV	- Inhibit expression of HLA class I and II antigens and antigen presentation, thus activation of T-cells	[34]
- EBV (EBNA-1)	- Cells expressing EBNA-1 do not present sufficient quantities of EBNA1 peptide on MHC class I.	[26]

Information has been reported on possible roles of HHV-6 in leukemia development (reviewed in [35]). HHV-6 was first isolated from patients with lymphoproliferative disorder. Arbuckle *et al*. [36] showed that HHV-6 antibody level was increased in children with ALL, but a subsequent study showed no significant difference (50 patients/50 controls). Slight but significant serological correlation was shown in case control studies with AML. The presence of HHV-6 IgM in 40% of children with leukemia, and high avidity of IgG compared with controls was shown in further studies. HHV-6 DNA was found in bone marrow cells of children with T-cell ALL. Importantly, a direct transforming ability of HHV-6 was shown *in vitro* with 3T3 cells and human epidermal keratinocytes. HHV-6 also has a unique ability to integrate viral DNA into chromosomes. Chromosomal transmission of HHV-6 DNA was shown in ALL cells in culture (reviewed in [35]). Apart from integration into chromosomal DNA, HHV-6 protein products are also able to interfere with host protein functions. U95 binds to NF-kB, altering the pathway, and U14 and ORF-1 bind and inactivate p53 [30]. These interactions likely contribute to the oncogenic transformation of infected host cells. Overall, although the results of studies showing the presence of serum antibodies to HHV-6 or viral DNA remain controversial, there is a possibility that HHV-6 can act via chromosomal integration and/or by the direct transforming ability of the virus.

Antibodies against herpes simplex, another member of the *Herpesviridae*, were prevalent in children with ALL in Iran. Patients had higher values of antibodies against HSV1 IgG (82.2%) comparing to a control group of 90 age-sex matched healthy children (54.5%) [37]. Other infectious agents associated with childhood leukemia include hepatitis B virus, human T-cell lymphoma virus I, and parvovirus B19. Although association with certain infectious agents is known, analysis of the presence of exogenous genomes in leukemia cells suggests that a single transforming agent is unlikely to cause leukemia development [11]. The mechanism for cancer development will probably involve several factors, wherein infectious agents can act as triggering factors.

#### Lymphoma

HL can be divided into two subtypes, EBV-associated and non-EBV-associated HL. Approximately 40 − 45% of the cases can be attributed to EBV infections, with high prevalence found in children under the age of 10 years. This could be associated with primary infections in younger children, and to loss of immune surveillance in older adults [38-40]. Evidence of an associations of HL with EBV came from serological studies that showed elevated titers of anti-EBV antibodies in HL patients [39,40]. In addition, the presence of viral DNA was demonstrated in malignant HL or Reed-Sternberg cells, where it established latency type II through the expression of Epstein-Barr nuclear antigen 1 (EBNA1), latent membrane protein 1 (LMP1), and latent membrane protein 2A (LMP2A) proteins [40,41].

There are three clinical variant types of BL, endemic, sporadic, and HIV-associated types. In all cases, tumor cells are characterized by *c-myc* oncogene translocation and differing positivity for EBV [42]. Endemic BL comprises up to 75% of all childhood cancers in malaria endemic areas, and are EBV positive in almost 100% of the cases. HIV-associated BL patients are positive for EBV in one third of all cases [41,42]. This finding suggests that BL is a polymicrobial disease [43], where malaria or HIV antigens act as co-agents in BL pathogenesis [44].

Although mucosa-associated lymphoid tissue (MALT) NHL occurs very rarely in children, it is noteworthy that its development is also associated with infection. The presence of *H. pylori* is demonstrated in almost all cases of MALT lymphoma [39,40,45]. Approximately half of the reported pediatric MALT lymphoma cases were observed in HIV positive patients [46,47]. Eradication of *H. pylori* with antibacterial therapy could lead to eradication and/or remission of the associated malignancy in most adult and pediatric cases [39,40,45,46].

#### Nervous system tumors

Nervous system tumors occur frequently in children, especially CNS tumor that is the second most common pediatric cancer. There are many types of nervous system tumors, and they occur at different ages. Neuroblastoma can be diagnosed prenatally or during the first 3 months of life. Astrocytomas are often diagnosed at 5 to 13 years of age, while primitive neuroectodermal tumors (PNET) and ependymomas are common at 3 years of age, and decline as age increases [48,49]. However, there is no known cause of nervous system cancer. Whether there is a genetic polymorphism which is crucial in a particular tumor type, or an involvement of toxins that regulate cell types at different stages of development remains unknown [50]. The very early onset of the disease suggests that parental factors may play a role during the period prior to conception, during gestation, or at birth [51]. It was reported that the children of mothers who had a documented viral infection during pregnancy had an 11-fold increased risk of having a malignant nervous system tumor. The infections linked to risk included rubella, mumps, varicella zoster, influenza, and herpes and respiratory infections [52,53].

Polyomaviruses Simian virus 40 (SV40), John Cunningham virus (JCV), and BK are suspected to cause various oncological malignancies. These viruses were introduced into the human population in the 1950s [54] and SV40 was suspected to cause brain tumors [55]. Later retrospective studies found no correlation of SV40 contaminated vaccines with brain cancer [56,57]. However, later reports found that the viruses were positively correlated with CNS tumors in adults and children [58,59].

The presence of JCV in pediatric tumors was detected by immunohistochemistry, PCR, and Southern blot, using T antigen and agnoprotein markers, but different laboratories found a discrepancy in results [60]. In 62 cases of various pediatric CNS tumors (medulloblastoma, ependymoma, choroid plexus papilloma, and pilocytic astrocytoma) the viral DNA was found in 28% of the ependymomas, and in 20% of the choroid plexus papillomas [61]. In a 9-year-old patient diagnosed with pleomorphic xanthoastrocytoma, the genomic sequences of JCV *LT, R*, and *VP1* were detected [62]. Further cases of JCV in pediatric pleomorphic xanthoastrocytoma were not reported.

A member of polyomaviruses BK is implicated in nervous system carcinogenesis. The virus is expressed in neuroblastomas, but not in normal adrenal medulla, and it colocalizes and binds to p53 [33]. The carcinogenic mechanisms and properties of polyomaviruses (Table 1) identify them as at least acting as cofactors in carcinogenesis, especially in children who have immature immune systems.

A growing number of studies on the herpes family of viruses and on childhood CNS tumors show positive correlations. Cytomegalovirus (CMV) was associated with non-CNS cancers and CNS tumors, including medulloblastoma, which is the most common CNS malignancy in children. CMV DNA and proteins were detected in primary medulloblastoma, in cell lines, and in xenografts. Thirty-seven primary medulloblastoma cases were examined, and 34 (92%) expressed immediate-early proteins, and 27 (73%) expressed late viral proteins [63]. The virus and viral particles were also detected in glioblastoma multiforme (GBM), a rare tumor among children [64]. Many CMV viral proteins inhibit apoptosis in infected cells. At least six different proteins were associated with inhibition of apoptosis and could therefore enhance survival of CMV-infected tumor cells [27,28]. It was reported that UL36 protein is responsible for chemotherapy resistance of infected cells in vitro [29]. CMV was also found to inhibit expression of HLA class I and class II antigens and antigen presentation, and thus activation of T-cells. CMV can also inhibit NK cell activation and cytotoxicity [34].

Recently, the mechanisms of how particular infectious agents contribute to carcinogenesis of CNS tumors were discussed. Infections can include not only viruses, but also bacteria and parasites [65]. It is obvious that adult CNS tumors are different from childhood tumors. However, a scarcity of studies on the association of childhood nervous sytem tumors with infectious agents limits the understanding of the role of infectious agents. Therefore, studies of how and to what extent viruses, bacteria, and parasites can contribute to the development of childhood tumors are needed.

#### Rare cancers

There are many other types of benign and malignant tumors which occur rarely in children. A case study of a child diagnosed with both sialoblastoma and hepatoblastoma demonstrated elevated levels of procalcitonin, proinflammatory stimulus to bacteria, and increased levels of C reactive protein, suggesting a response against bacterial infections [66]. Sialoblastoma affects parotid glands during infancy, and is rare, with only 24 cases reported in MedLine (1990–2008). Hepatoblastoma originates in the liver, and comprises less than 1% of reported tumors among children from infancy to 3 years of age [67]. Another liver tumor, a hepatocellular carcinoma (HCC), is also rare in children [68]. Hepatitis B virus (HBV) was found to be responsible for HCC in children in Taiwan, where HBV was endemic [69]. This relationship of HBV and childhood cancer was established as a result of discovering 100% HBsAg seropositivity among children with HCC and HBV DNA in their neoplasms [69,70]. The role of HBV infection in the cause of hepatocellular carcinoma is further confirmed when Taiwanese HBV immunization programme significantly reduced the cancer incidence in vaccinated children compared to non-vaccinated children [71]. In addition to this, the twenty-year study has shown lower incidence of HCC in those Thai children who received HBV vaccine at birth [72].

Another infrequent type of childhood cancer is head and neck cancers which include lesions on the lip, oral cavity, nose and paranasal sinuses, naso-pharynx, oropharynx, hypopharynx, and larynx [73]. It accounts for 5% of all childhood cancers [74]. Syrjanen *et al*. [75] first reported the association of human papilloma virus (HPV) infections in the oncogenesis of laryngeal and oral cancers by observing similar morphological features both in oral and cervical squamous cell lesions. A direct oncogenic role of high risk HPV E6 and E7 proteins was identified. The viral proteins bind and thereby inactivate the tumor suppressors [32]. HPV probably employs the same mechanism of cancer development in different age groups, including children, and may act as an independent risk factor for oral cancer, another extremely rare childhood cancer [76].

To continue, nasopharyngeal carcinoma (NPC) is a rare cancer which occurs on the epithelium of the nasopharynx and accounts for about 1% of all childhood tumors. According to WHO there are three subtypes of NPC, including squamous cell carcinoma commonly found in adults, non-keratinizing carcinoma, and undifferentiated carcinoma. Childhood cancer cases are usually the latter subtype and EBV is involved in its aetiology [77,78]. It was found that abundance of LMP1 expression of EBV protein correlated with a patient’s age. There were higher levels of LMP1 in younger patients’ specimens in Tunisia (n = 22 out of 82) [79]. It was in concordance with previous findings, where anti-viral capsid antigen, early antigen IgG and IgA levels were lower in juvenile form of NPC compared to adult form [80,81]. NPC incidence is 1 per 100,000 children diagnosed annually in the US whilst it is more common in children of the Southeast Asia and Northern African region, with 8–25 per 100,000 cases recorded annually [82,83]. In Hong Kong 80% of children are infected by 6 years of age and almost 100% have seroconverted by the age of 10 years [84]. The disclosure of the EBV nuclear antigen and viral DNA in the NPC demonstrated that EBV can infect epithelial cells and is associated with their malignant transformation.

Kaposi’s sarcoma (KS) is a tumor, which originates from the cell lining of lymph or blood vessels. It is classified primarily into three types: classical, endemic and epidemic. Classical KS is prevalent among elderly men; whilst younger generation, located close to African equator, is prone to endemic KS, or African KS. Epidemic KS occurs in patients with AIDS caused by HIV [85]. It is to be noted that before the onset of HIV in 1985 KS had been endemic in Africa [86]. It was reported that KS is caused by Human Herpes Virus 8 (HHV-8, also known as Kaposi sarcoma-associated herpesvirus) in children whose immune system is weakened [87,88]. During the period of AIDS infection the cases of childhood KS increased more than 40-fold [89]. Prevalence of KS incidence among children is higher in Africa, where both HHV-8 and HIV are endemic, in contrast to well-resourced states, where patients have access to antiretroviral therapy [87,90].

To sum up, the above-mentioned cancers are rare or even extremely rare in children, and more studies are needed to establish the mechanism of infection-based cancer development. However, there is still space to suspect the pathogens, which stimulate these rare malignancies in young generation.

### Possible mechanisms of childhood cancers

The mechanisms of infectious etiologies of CC have been studied since the 1970s [91,92], yet a definitive association has not been established for the majority of childhood cancers. It is known that viruses such as CMV, HPV, VZV, and HSV can cross the placenta and infect the fetus. Various infections can also be acquired during passage through the birth canal if the mother is infected, or after admission to the nursery. It was also observed that HPV and EBV are sometimes found in breast milk and can be transmitted to the infant through breastfeeding [93]. Some of the above mentioned viruses are also found to cause tumors in adults (i.e., HPV in the cervix and in anogenital cancers, human HHV-8 in Kaposi’s sarcoma, and EBV in Burkitt’s lymphoma). Many mechanisms are used by infectious agents to survive in a host and create favorable conditions for neoplastic transformation. It is also possible that a child can inherit mutations that occurred in the germ cells of the parents via viral mutagenesis. Partial sequences of HHV-6A have been found in members of the same families [36]. All of the mechanisms listed in Table 1 could be exploited in combination, synergistically creating favorable conditions for neoplastic lesions to develop. Disruption of tumor suppression, in combination with apoptosis, immune evasion, and low-grade inflammation could enhance the accumulation of mutations, and this would select for preneoplastic cells, which over time could lead to the development of a tumor. Infants are born with an undeveloped immune system, and the prevalence of latent viral infections in adults that come in contact with newborns could facilitate the transmission of viruses. Thus, the chance of latent infections is likely.

### Hypothesis

There are several proposed theories for childhood cancer development. Most theories focus on leukemia progression. One of them is the Greaves’ hypothesis of a “delayed infection”. It states that a child’s immature immune system requires early exposure to common infections for the proper immune system maturation. The lack of it leads to the aberrant response to infection. As evidence for the hypothesis, there are examples of well protected babies from developed countries (hygienic conditions), and they prone to get childhood leukemia i.e. they had aberrant response to delayed exposure to infectious agents [94]. Another hypothesis, the Kinlen hypothesis, proposes that a common infectious agent is responsible for increased leukemia cases transmitted by adults during a large-scale rural–urban population mixing [95]. Both Greaves and Kinlen hypothesis are supported by epidemiological data which accumulated over the past 15 years. They propose an unknown infectious agent (mostly viral by Kinlen) that trigger an aberrant immune response and thus can be a causative agent of childhood ALL. A more recent unifying hypothesis, of “cell transformation by replication-defective mutants,” suggests that only viruses which have defects in replication machinery show elevated transformation potential, and when they are present in cells with certain chromosomal translocations, it can lead to leukemia development [96]. Finally, the “infective lymphoid recovery” hypothesis proposes that infective stress, triggering the heat shock response in infancy, stimulates proinflammatory cytokines and inhibits apoptosis. This, in turn, leads to a decline in antitumor immunity and in B-cell maturation arrest. This hypothesis addresses the infection paradox stated in Greaves’ and Kinlen’s hypotheses, that an unhygienic environment primes the adaptive immune response and is protective against childhood ALL, while multiple infections occurring later increase the risk of childhood ALL [97]. Overall, it is proposed that infection can be a triggering mechanism, and most likely, the immunological state, genetic alterations, and infections have a cumulative effect on leukemia development, and possibly on the development of other cancer types.

## Conclusions

The etiology of childhood cancers still remains unknown. In addition to genetic and environmental factors, we have shown that infectious agents also play a significant role in cancer development. These include human herpesviruses (especially EBV, CMV, and HHV-6), polyomaviruses (SV40, JCV, and BKV), HPV, HIV, HCV, and *H. pylori*. These infectious agents can be controlled by treatment and/or vaccination. It was shown that, using immune globulins during pregnancy and after birth, some protective effect against some herpes viruses and HBV [98-100] was observed. Vaccines may also protect against mother-to-child transmission of infections [101]. Because the elimination of infection could minimize the risk of childhood cancer, everything possible should be done to prevent or control infections in both the mother and child. Sanitary measures and regulations can help to reduce the occurrence of such infections during pregnancy, the first years of life, and in healthcare institutions. Early diagnosis should also be improved and included in family planning programs. If the infections are already present or at high risk, more treatments should be done to prevent mother-to-child transmission, with specific attention focused on the development of safer vaccines and therapeutics [102]. Information on possible risks and proper counseling should be available to susceptible populations. However, there is still insufficient clinical data to provide a complete plan to reduce the risks of infection-driven cancers in children.

## Competing interests

The authors declare they have no competing interests.

## Authors’ contributions

KA, AM, AK, NK, SZ, AD, BA, and YB performed the literature research, composed the article and approved the final version to be submitted. All authors read and approved the final manuscript.
